# Chemical fingerprinting and quantitative constituent analysis of Siwu decoction categorized formulae by UPLC-QTOF/MS/MS and HPLC-DAD

**DOI:** 10.1186/1749-8546-8-5

**Published:** 2013-03-01

**Authors:** Shulan Su, Wenxia Cui, Wei Zhou, Jin-ao Duan, Erxin Shang, Yuping Tang

**Affiliations:** 1 Jiangsu Key Laboratory for TCM Formulae Research, Nanjing University of Chinese Medicine, Nanjing, 210023, PR China; 2 College of Pharmacy, Jiangsu University, Zhenjiang, 212013, PR China

## Abstract

**Background:**

Siwu decoction categorized formulae (SWDCF) are widely used for treating gynecological diseases. This study aims to elucidate the differences of bioactive constituents in SWDCF by ultra-high performance liquid chromatography coupled with time-of-flight mass spectrometry (UPLC - QTOF - MS /MS) and HPLC-DAD.

**Methods:**

An efficient method based on UPLC - QTOF - MS /MS was developed for identifying the chemical profiles of SWDCF. HPLC-DAD method was used for quantifying seven chemical markers in SWDCF.

**Results:**

Eighty four components were identified or characterized, including ten organic acids, thirty glycosides (monoterpene or iridoid or phenylpropanoids glycosides), fourteen lactones, eighteen flavonoids, and eleven alkaloids in the complex system. The datasets of t*R*-*m/z* pairs, ion intensities and sample codes were processed with supervised orthogonal partial least squared discriminant analysis to compare these decoction samples. After a clear classification was established, OPLS-DA was performed and 16 common components with relative quantity in SWDCF samples were determined. Gallic acid, protocatechuic acid, vanillic acid, caffeic acid, paeoniflorin, ferulic acid, and senkyunolide I were selected as the chemical markers to identify SWDCF by HPLC-DAD.

**Conclusion:**

The chemical profiles with 84 components in SWDCF, including monoterpene glycosides, acetophenones, galloyl glucoses, even some isomers in the complex system were characterized by UPLC–QTOF–MS/MS.

## Background

Chinese medicine (CM) is holistically formulated for treating complicated CM syndromes (“*ZHENG*” in Chinese) [1]. The researches on categorized formulae (CF) based on a basic formula composition may help understand the rules of formulation and *Fang-Zheng* consistency in CM [2].

Siwu decoction (SWD) is a classical prescription that is widely used for the treatment of women’s diseases in CM, such as relief of emmeniopathy, climacteric syndrome, dysmenorrhea and other estrogen-related diseases [3]. A recent study demonstrated that the SWD can be synergistically used with Western medicine [4]. SWD consists of four herbs, *i.e.*, *Angelicae sinensis Radix*, *Chuanxiong Rhizoma*, *Paeoniae Radix Alba*, and *Rehmanniae Radix*[5]. A series of SWD-based formulae used to treat women’s diseases especially for primary dysmenorrheal (PD) are considered as Siwu decoction categorized formulae (SWDCF), including Taohong Siwu decoction (THSWD), Xiangfu Siwu decoction (XFSWD), Shaofu Zhuyu decoction (SFZYD), and Qinlian Siwu decoction (QLSWD). The compositions and applications of SWDCF were described in Table 1. These formulae are usually adopted to treat different symptoms of different kinds of PD.

**Table 1:** The composition and application of SWDCF

**Prescriptions**	**Composition**	**Application**
Siwu decoction (SWD)	(*Angelica sinensis*) Angelicae sinensis Radix 9 g, (*Ligustium chuanxiong*) Chuanxiong Rhizoma 9 g, (*Paeonia lactiflora*) Paeoniae Radix Alba 9 g, and (*Rehmannia glutinosa*) Rehmanniae Radix 9 g	Cure women’ diseases, such as dysmenorrhea and other estrogen related diseases
Taohong-Siwu decoction (THSWD)	(*Angelica sinensis*) Angelicae sinensis Radix 9 g, (*Ligustium chuanxiong*) Chuanxiong Rhizoma 9 g, (*Paeonia lactiflora*) Paeoniae Radix Alba 9 g, (*Rehmannia glutinosa*) Rehmanniae Radix 9 g, (*Prunus persica*) Persicae Semen 9 g, and (*Carthamus tinctorius*) Carthami Flos 6 g	Cure PD with syndrome of deficiency of blood.
Xiangfu-Siwu decoction (XFSWD)	(*Angelica sinensis*) Angelicae sinensis Radix 9 g, (*Ligustium chuanxiong*) Chuanxiong Rhizoma 4.5 g, (*Paeonia lactiflora*) Paeoniae Radix Alba 4.5 g, (*Rehmannia glutinosa*) Rehmanniae Radix 12 g, (*Cyperus rotundus*) Cyperi Rhizoma, 4.5 g, (*Corydalis yanhusuo*) Corydalis Rhizoma 4.5 g, and (*Aucklandia lappa*) Aucklandiae Radix 3 g	Cure PD induced by stagnation of the circulation of vital energy
Shaofu-Zhuyu decoction (SFZYD)	(*Angelica sinensis*) Angelicae sinensis Radix 9 g, (*Ligustium chuanxiong*) Chuanxiong Rhizoma 3 g, (*Corydalis yanhusuo*)Corydalis Rhizoma 3 g, (*Paeonia veitchii*) Paeoniae Radix Rubra 6 g, (*Cinnamomum cassia*) Cinnamomi Cortex 3 g, (*Foeniculum vulgare*) Foeniculi Fructus 1.5 g, (*Zingiber officinale*) Zingiberis Rhizoma 3 g, (*Commiphora myrrha*) Myrrha 3 g, (*Trogopterprus xanthipes*) Trogopterpri Faeces 6 g, and (*Typha angustifolia*) Typhae Pollen 9 g	Cure PD with syndrome of cold coagulation and blood stasis
Qinlian-Siwu decoction (QLSWD)	(*Angelica sinensis*) Angelicae sinensis Radix 9 g, (*Ligustium chuanxiong*) Chuanxiong Rhizoma 9 g, (*Paeonia lactiflora*) Paeoniae Radix Alba 9 g, and (*Rehmannia glutinosa*) Rehmanniae Radix 9 g, (*Scutellaria baicalensis*) Scutellariae Radix 4.5 g and (*Coptis chinensis*) Coptidis Rhizoma 4.5 g	Cure PD resulted from pathogenic heat or inflammation

Our recent studies [6,7] showed that SWDCF inhibited uterine contraction and had analgesic effects on primary dysmenorrhea model mice. Their anti-inflammatory activities, hemorheological improvement and ovarian regulation in rats with blood stasis were elucidated [8,9]. Moreover, SWDCF inhibited COX-2 enzyme and platelet aggregation *in vitro*[9,10]. The main constituents of SWDCF belong to several natural product groups, such as phenolic acids, phthalides, alkaloids, terpene glycosides, iridoid glycosides, flavones, and *etc.*[10-13]. However, no report on the constituent profiles of SWDCF is available.

The investigations of the components of SWDCF are important to reveal their effects and action mechanisms. However, the complicated chemical profiles of SWDCF components demand a rapid and efficient method for chemical profiling of SWDCF.

This study aims to profile the constituents of SWDCF by UPLC-QTOF-MS/MS with MarkerLynx analysis and identify SWDCF with several chemical markers.

## Methods

### Chemicals, reagents and materials

**Figure 1:**
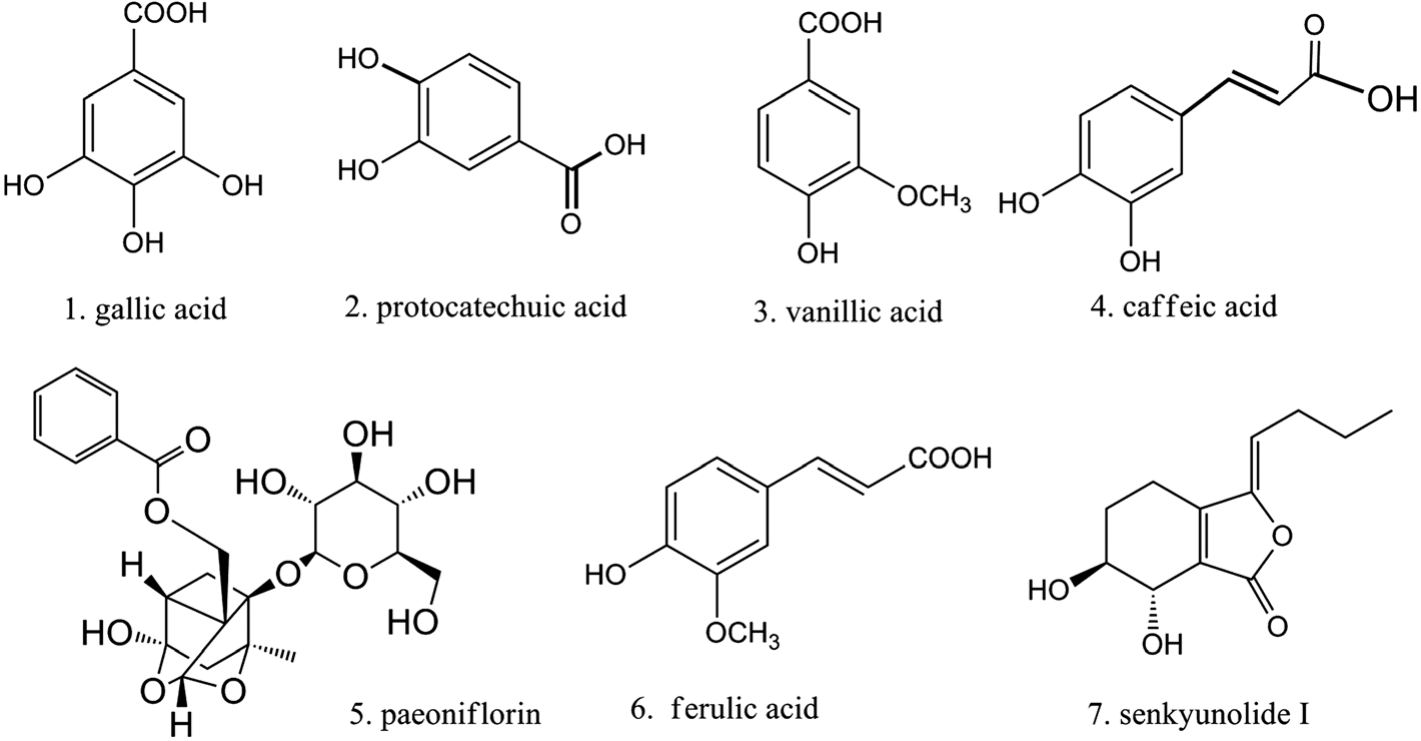
Chemical structures of the quantitative compounds in the Siwu decoction categorized formulae.

Gallic acid, protocatechuic acid, vanillic acid, caffeic acid, paeoniflorin, ferulic acid, and senkyunolide I (Figure 1) were purchased from National Institute for the Control of Pharmaceutical and Biological products (China).

Acetonitrile was HPLC-grade from Merck (Darmstadt, Germany) and deionized water was purified by a Millipore water purification system (Millipore, Milford, MA, USA) and filtered with 0.22 μm membranes. Other reagent solutions were of analytical grade (Sinopharm Chemical Reagent Co., Ltd., Shanghai, China).

Herbal medicines of *Angelicae sinensis Radix*, *Chuanxiong Rhizoma, Paeoniae Radix Alba, Rehmanniae Radix, Persicae Semen, Carthami Flos, Cyperi Rhizoma, Aucklandiae Radix, Corydalis Rhizoma, Paeoniae Radix Rubra, Cinnamomi Cortex, Foeniculi Fructus, Zingiberis Rhizoma, Myrrha, Trogopterpri Faeces, Typhae Pollen, Scutellariae Radix and Coptidis Rhizoma,* were purchased from the following towns (provinces): *Minxian* (*Gansu*), *Pengzhou* (*Sichuan*), *Tongling* (*Anhui*), *Huaiqing* (*Hehan*), *Anguo* (*Hebei*), *Tacheng* (*Xinjiang*), *Linyi* (*Shandong*), *Lijiang* (*Yunnan*), *Songyang* (*Zhejiang*), *Chifeng* (*Neimeng*), *Yulin* (*Guangsi*), *Wuwei* (*Gansu*), *Yulin* (*Guangsi*), *Guangdong*, *Changzhi* (*Shanxi*), *Yixing* (*Jiangsu*), *Chengde* (*Hebei*), and *Mianyang* (*Sichuan*), respectively. All crude herbs were identified by the Prof. Jin-ao Duan in accordance with the Pharmacopoeia of People’s Republic of China [14]. The voucher specimens (no. NJUTCM-20101112-20101129) were deposited in Jiangsu Key Laboratory for TCM Formulae Research, Nanjing University of Chinese Medicine.

### Apparatus and chromatographic conditions

#### UPLC-QTOF-MS/MS qualitative analysis

Chromatography was performed on an AcQuity™ UPLC system with a conditioned autosampler (Waters Corp., Milford, MA, USA) at 4°C. The separation was carried out on an AcQuity UPLCTM BEH C_18_ column (100 mm × 2.1 mm i.d., 1.7 μm; Waters Corp., Milford, MA, USA) maintained at 35°C. The mobile phase consisted of 0.1% formic acid (HCOOH) in water as solvent A and acetonitrile (ACN) as solvent B. The gradient conditions of the mobile phase were: 0 min 95% A, 9.0 min 56% A, 12.0 min 26% A, 20.0 min 10% A, 22.0 min 10% A, 25.0 min 95% A. The flow rate was 0.40 mL/min. The sample injection volume was 5 μL.

Mass spectrometric detection was carried out on an AcQuity Synapt Mass Spectrometer equipped with an electrospray ionization (ESI) interface (Waters, Milford, MA, USA). High purity nitrogen was used as the nebulizer and auxiliary gas; argon was utilized as the collision gas. The ESI source was operated in positive and negative ionization mode with a capillary voltage of 3 kV, sampling cone voltage of 10 V, cone gas flow of 50 L/h, desolvation gas flow of 700 L/h, desolvation temperature of 350°C, source temperature of 120°C, collision energy of 45 V, and the full scan spectra from 100 to 1000 Da. Leucine-enkephalin was used as the lock mass generating an [M + H]^+^ ion (*m/z* 556.2771) and [M-H]^-^ ion (*m/z* 554.2615) at a concentration of 200 pg/mL and flow rate of 100 μL/min. Data acquisition and processing were performed by MassLynx 4.1 and MarkerLynx 4.1 (Waters Corp., Milford, MA, USA) for peak detection.

#### HPLC-PDA quantificative analysis

The quantification analysis was performed on a Waters-2695 Alliance HPLC (Waters Corporation, Milford, MA, USA) equipped with an on-line degasser, an auto-sampler and a 2996 photodiode array detector. UV detection was achieved at 210–400 nm. A Waters Sun Fire™ C_18_ column (4.6 × 250 mm, 5 μm, serial no. 186002560 Waters Corporation, USA) was used. A linear gradient elution of A CH_3_OH and B (CH_3_COOH: H_2_O = 0.1: 100) was used. The gradient program is 85% B in 0–8 min, 85-80% B in 8–10 min, 80-74% B in 10–30 min, 74-66% B in 30–40 min, 66-34% B in 40–60 min, 34-10% B in 60–85 min. The solvent flow rate was 1 mL/min and the column temperature was set at 30°C. Re-equilibration duration was 15 min between individual runs. A Waters 2996 photo diode array was connected to the liquid chromatography for detection of the raw data.

### Preparation of standard solutions

A mixed standard stock solution containing gallic acid (**1**), protocatechuic acid (**2**), vanillic acid (**3**), caffeic acid (**4**), paeoniflorin (**5**), ferulic acid (**6**), and senkyunolide I (**7**) was prepared in methanol. The working standard solutions were prepared by diluting the mixed standard solution with methanol to a series of proper concentrations within the ranges: **1**, 148–1480.0 μg/mL; **2**, 38.40–384.00 μg/mL; **3**, 32.0-320.0 μg/mL; **4**, 35.6-356.0 μg/mL; **5**, 476.0-4760.0 μg/mL; **6**, 136.0-1360.0 μg/mL; **7**, 46.0-460.0 μg/mL. The standard stock and working solutions were all stored at 4°C and filtered through a 0.22 μm membrane prior to injection.

### Preparation of sample solutions

The mixtures of SWDCF (Table 1) were crushed into small pieces and refluxed with 10 times water for 2 h twice. The filtrates from each decoction were combined and concentrated to 1.0 mg/mL at 70°C. The filtrates were added 95% ethanol until the concentration of ethanol was adjusted to 50%. After centrifugation at 3000 × *g* for 10 min, the supernatant was stored at 4°C and filtered through a 0.22 μm membrane filter before the UPLC-QTOF-MS analysis and the HPLC-DAD analysis.

### Validation of the HPLC method

#### Calibration curves, limits of detection and quantification

The working standard solutions with at least six different concentrations (**1**, 148–1480.0 μg/mL; **2**, 38.40-384.00 μg/mL; **3**, 32.0-320.0 μg/mL; **4**, 35.6-356.0 μg/mL; **5**, 476.0-4760.0 μg/mL; **6**, 136.0-1360.0 μg/mL; **7**, 46.0-460.0 μg/mL) were analyzed, and the calibration curves were calculated by linear regression of the double logarithmic plots of the peak area versus the concentration of the reference solution injected. The limits of detection and quantification (LODs and LOQs) under the present chromatographic conditions in this study were determined by diluting the standard solution when the signal-to-noise ratios (S/N) of analytes were about 3 and 10, respectively. The S/N was calculated as the peak height divided by the background noise value.

### Precision, repeatability and accuracy

The intra-day and inter-day variations, which were chosen to determine the precision of the developed method, were investigated by determining the seven analytes in six replicates during a single day and by duplicating the experiments on three consecutive days. Variations of the peak area were taken as the measures of precision and expressed as relative standard deviations (R.S.D.).

Repeatability was confirmed with six independent analytical sample solutions prepared according to the methods describing before and expressed by R.S.D. These SWTCF sample solutions was stored at 4°C, and injected into the HPLC apparatus at 0, 2, 4, 8, and 12 hr, respectively, to evaluate the stability of the solution.

Recovery test was performed by adding accurate amounts of the seven standards into a certain amounts separately to evaluate the accuracy of this method. The spiked samples were then extracted, processed, and quantified in accordance with the methods mentioned above. Six replicates were performed for the test. The average recovery percentage was calculated by the formula: recovery (%) = (observed amount − original amount)/spiked amount × 100%.

### MarkerLynx™ analysis

The data obtained from positive and negative ion mode of all determined samples were analyzed by MarkerLynx v4.1 software (Waters, Manchester, UK) with the help of multivariate statistical analysis, to reveal any potential changed components in SWDCF. The original data were processed using the following parameters: initial retention time of 0 min, final retention time of 25 min, and mass in the range 100–1000 Da, with a mass tolerance of 0.02 Da, mass window of 0.02 Da, retention time window of 0.1 min, noise elimination level 6. For peak integration, peak width at 5% of the height was 1 s, peak-to-peak baseline noise was automatically calculated, and peak intensity threshold was 10. No specific mass or adduct was excluded. Isotopic peaks were excluded for analysis. For data analysis, a list of the intensities of the peaks detected was generated using t*R* and mass data (*m*/*z*) pairs as the identifier of each peak. An ID was assigned to each of these t*R*-*m*/*z* pairs in the order of their UPLC elution for data alignment [15]. The process was repeated for each run. After completion, the correct peak intensity data for each t*R*-*m/z* pair of the entire batch of samples were aligned in the final data table. The ions that showed the same t*R* (with a tolerance of 0.1 min) and *m/z* value (with a tolerance of 0.05 Da) in different samples were considered as the same ion. For those peaks hard to be detected in the sample, the ion intensities were documented as zero in the final data table. Before submitted for multivariate analyses, the ion intensities for each detected peak were normalized against the sum of the peak intensities. The resulting three-dimensional data comprising of peak number (t*R*-*m/z* pair), sample name and ion intensity were analyzed by OPLS-DA with the MarkerLynx software.

## Results and discussion

### Identification of SWDCF constituents by UPLC-QTOF-MS/MS

The multiple chemical components in the SWDCF samples were identified and characterized by both negative and positive ESI modes. The total current chromatograms at the two modes were shown in Additional file 1: Figures S1-1 and 1-2. Diagnostic fragmentations were then checked by MS/MS to confirm the results. Eighty-four constituents were identified by comparing the t*R*, UV_λmax_, and MS fragments characteristics of the compounds. The analyzed and identified compounds were listed in Table 2. Among these 84 compounds, there were ten organic acids, thirty glycosides (monoterpene or iridoid glycosides or phenylpropanoids), fourteen lactones, eighteen flavonoids, and eleven alkaloids.

**Table 2:** Characterization of compounds in Siwu decoction categorized formulae by UPLC-QTOF-MS/MS

**Peak no.**	**T**_**R **_**(min)**	**Positive ions ( *m/z *)**	**Negative ions ( *m/z *)**	**λ**_**max **_**(nm)**	**Identification**	**Origins**
1	1.42	171 [M + H]^+^	169 [M-H]^-^, 125 [M-HCOO]^-^, 97 [M-H-CO_2_-CO]^-^	216, 270	Gallic acid	A, B, C, D, E
2	3.07	355 [M + H]^+^, 377 [M + Na]^+^	353 [M-H]^-^, 191[M-C_9_H_7_O_3_]^-^, 179[M-C_7_H_11_O_5_]^-^, 173 [M-C_9_H_7_O_3_-H_2_O]^-^	326	Chlorogenic acid	A, B, C, D, E
3	4.10	181 [M + H]^+^	179 [M-H]^-^, 135 [M-HCOO]^-^	320, 240	Caffeic acid	A, B, C, D, E
4	3.52	169 [M + H]^+^	167 [M-H]^-^, 153 [M-CH_3_]^-^, 137 [M-OCH_3_]^-^, 123 [M-HCOO]^-^	290	Vanillic acid	A, B, C, D, E
5	4.90	195 [M + H]^+^	193 [M-H]^-^, 178 [M-H-CH_3_]^-^, 149 [M-H-CO_2_]^-^, 134 [M-HCOO]^-^	320	Ferulic acid	A, B, C, D, E
6	8.90	195 [M + H]^+^	193 [M-H]^-^, 237 [M-H + HCOO]^-^, 179 [M-CH_3_]^-^	310	Isoferulic acid	A, B, C, D, E
7	4.68	-	153 [M-H]^-^, 141 [M-H-CH_3_]^-^, 109 [M-H-CO_2_]^-^	310	Protocatechuic acid	A, B, C, D, E
8	9.44	-	163 [M-H]^-^, 117 [M-H-HCOO]^-^		Coumaric acid	A, B
9	2.98	139 [M + H]^+^, 161 [M + Na]^+^	137 [M-H]^-^, 119 [M-H-H_2_O]^-^		*p*-hydroxy benzoic acid	A, B
10	7.02	-	121 [M-H]^-^	238, 272	Benzoic acid	A, B
11	1.47	361 [M + H]^+^	359 [M-H]^-^, 493 [M-H + HCOO]^-^, 405 [M + HCOO]^-^, 197 [M-H-glu]^-^, 179 [M-H-glu-H_2_O]^-^	210, 270	1-*O*-*β*-D- glucopyranosyl -paeonisuffrone	A, B
12	1.51	517 [M + Na]^+^	493 [M-H]^-^, 457 [M-H-2H_2_O]^-^, 443 [M-H-2H_2_O- CH_3_]^-^, 331 [M-2H-glu]^-^, 169 [M-sucrose]^-^	220, 270	1^′^-*O*-galloylsucrose	A
13	2.89	562 [M + Na]^+^, 383 [M + H-glu]^+^, 261[M + H-glu-benzoyl]^+^, 197[M + H-glu-benzoyl-SO_2_]^+^	543[M-H]^-^, 495[M-CH_2_OH-H_2_O]^-^, 461[M-SO_2_-H_2_O-H]^-^, 341[M-SO_2_-C_7_H_5_O-2OH]^-^, 243[M-glu-benzoyl-H_2_O-CH_3_]^-^	232	Paeoniflorin sulfonate	A, B
14	3.85	481 [M + H]^+^, 319 [M + H - glu]^+^, 197 [M + H -glu-benzoyl]^+^, 161 [M + H -glu-benzoyl-2H_2_O]^+^, 133 [M + H -glu-benzoyl-2H_2_O-CO]^+^	479 [M-H]^-^, 525 [M + HCOO]^-^, 416 [M-CH_2_OH-CH_3_-H_2_O]^-^, 283[M- glu-H_2_O-OH]^-^, 177[M-glu-benzoyl-2H_2_O]^-^	233	Albiflorin	A, B, C, D, E
15	4.29	-	695[M-H]^-^, 631[M-SO_2_-H]^-^, 525[M-galloyl-H_2_O]^-^, 519[M-benzoyl-4H_2_O]^-^, 479[M-galloyl-SO_2_]^-^, 449[M-galloyl-SO_2_-CO-H]^-^	275	Galloylpaeoniflorin sulfonate	A, B
16	5.84	481 [M + H]^+^, 503 [M + Na]^+^, 397, 319, 197	479[M-H]^-^, 525[M + HCOO]^-^, 449[M-CH_2_OH]^-^, 416[M-CH_2_OH-CH_3_-H_2_O]^-^, 327[M-benzoyl-CH_2_OH-CH_3_-2H]^-^, 176[M-benzoyl-glu-2H_2_O-H]^-^,121[benzoyl]^-^	230	Paeoniflorin	A, B, C, D, E
17	4.36	497 [M + H]^+^, 519 [M + Na]^+^, 381 [M + Na–pOHBA]^+^, 357 [M + Na–Glc]^+^, 323 [M + Na–aglycone]^+^, 271 [M + Na–C_13_H_12_O_5_]^+^	495[M-H]^-^, 449[M-CH_3_-CH_2_OH-H]^-^, 479[M-OH]^-^, 327[M-p-hydroxybenzoyl-CH_2_OH-OH]^-^	235, 322	Oxypaeoniflorin	A
18	3.91	481 [M + H]^+^, 503 [M + Na]^+^	479 [M-H]^-^, 525[M + HCOO]^-^, 449[M-CH_2_OH]^-^, 327[M-benzoyl-CH_2_OH-OH]^-^	220, 275	Isopaeoniflorin / albiflorin R1	A
19	4.97	633 [M + H]^+^, 655 [M + Na]^+^, 153[galloyl + H-H_2_O]^+^	631[M-H]^-^, 525[M-benzoyl-2H]^-^, 449[M-galloy-HCHO]^-^	275	Galloylpaeoniflorin	A, B
20	4.87	-	939 [M-H]^-^, 631 [M-2galloyl-3H]^-^, 469 [M- 2galloyl -3H-glu]^-^	220, 280	Pentagalloylglucose	A, B,C
21	5.20	-	623[M-H]^-^, 515[M-C_6_H_5_O_2_]^-^		Acteoside/isoacteoside / forsythoside A	A, B
22	5.78	481[M + H]^+^, 319[M + H-glu]^+^, 197[aglycone + H]^+^, 179[aglycone + H-H_2_O]^+^, 161[aglycone + H-2H_2_O]^+^, 133[aglycone + H-2H_2_O-CO]^+^	479[M-H]^-^, 463[M-OH]^-^, 341[M-benzoyl-2OH]^-^		Mudanpioside I	A, B
23	6.67		647[M-H]^-^, 513[M-benzoyl-HCHO]^-^, 507[M-benzoyl-2H_2_O]^-^, 391[M-2benzoyl-HCHO-OH]^-^		Benzoypaeoniflorin sulfonate	A, B
24	9.29	585[M + H]^+^, 607 [M + Na]^+^, 602[M + H_2_O], 463[M + H-benzoyl]^+^, 301[M + H-glu]^+^, 179[M + H-benzoyl-glu]^+^, 151[M + H-benzoyl-glu-CO]^+^	583[M-H]^-^, 629[M + HCOO]^-^	220, 270	Benzoylpaeoniflorin	A, C
25	8.09		583[M-H]^-^, 629[M + HCOO]^-^	220, 270	Isobenzoylpaeoniflorin	A, B
26	4.20	-	799[M-H]^-^, 637[M-caffeoyl]^-^, 525, 479, 449		cistanoside A or jionoside A1/A2	A
27	3.66	660[M + H_2_O], 625[M + H-H_2_O]^+^, 341[M + H-H_2_O-glu-benzoyl]^+^, 301[M + H-H_2_O-2glu]^+^	641[M-H]^-^, 687[M + HCOO]^-^, 611[M-CH_2_OH]^-^, 593[M-CH_2_OH-H_2_O]^-^, 341[maltosyl]^-^, 497[M-benzoyl-2H_2_O-4H]^-^	212, 277	Isomaltopaeoniflorin/6^′^-O-β-D-glucopyranosylalbiflorin	A
28	2.75	729[M + Na]^+^	705[M-H]^-^, 495, 443, 341, 271, 193	215	Isomaltopaeoniflorin sulfonate	A
29	4.14	463[M + H]^+^, 484[M + Na]^+^, 498, 301, 179	461[M-H]^-^, 525, 479, 449, 327	232	decaffeoyl-verbascoside	A
30	5.68	363[M + H]^+^, 385[M + Na]^+^, 340, 319, 197	361[M-H]^-^, 407[M + HCOO]^-^, 311, 287	232	6-*O*-*β*-D-glucopyranosyl lactinolide	A
31	1.40	687 [M + H]^+^	685[M-H]^-^, 731 [M-H + HCOO]^-^, 493 [M-H-glu- HCOO]^-^	215, 273	Rehmannioside D	A, B
32	7.18	525 [M + H]^+^, 207	523[M-H]^-^, 569 [M + HCOO]^-^, 407, 341, 183		Melittoside	A, B
33	3.71	-	785[M-H]^-^, 687[M-98]^-^	236, 279	Echinacoside	A
34	4.55		435[M + HCOO]^-^, 389[M-H]^-^, 327[M-3CH_3_-H_2_O]^-^, 178[M-C_13_H_24_O_2_]^-^	-	Rehmaionoside A/B	A, B
35	4.94	-	813[M-H]^-^, 515[M-C_6_H_12_O_4_-C_9_H_10_O_2_-H]^-^, 469[M-glu-feruloyl]^-^		Jionoside B1/B2	A, B
36	6.51	653[M + H]^+^	651[M-H]^-^, 505[M-rhamnosyl-H]^-^	276	Martynoside isomer	A
37	4.98	653[M + H]^+^	651[M-H]^-^, 445[M-ferulic acid-2CH_3_]^-^, 389[M-feruloyl-HCHO-3H_2_O-2H]^-^	235, 322	Martynoside	A
38	5.52	347[M + H]^+^	345[M-H]^-^, 391[M + HCOO]^-^, 183[M-gku]^-^, 179[M-C_10_H_15_O_2_]^-^	-	Rehmapicroside	A,E
39	7.21	207[M + Na]^+^	183[M-H]^-^, 165[M-H_2_O-H]^-^, 139[M-HCOO]^-^	-	Rehmapicrogenin	A,E
40	3.20	459[M + H]^+^, 325, 163	457[M-H]^-^, 323	215,237	Amygdalin	B
41	6.09	249[M + Na]^+^, 227[M + H]^+^, 209[M-H_2_O + H]^+^, 191[M-2H_2_O + H]^+^, 181[M-H_2_O-CO + H]^+^	225[M-H]^-^, 195, 125	277	Senkyunolide J	A, B, C, D,E
42	6.26	225[M + H]^+^, 247[M + Na]^+^, 207.1014[M-H_2_O + H]^+^, 189.0889[M + H-2H_2_O]^+^, 165.0890 [M + H-H_2_O-C_3_H_6_]^+^	223[M-H]^-^, 205, 177	270	Senkyunolide I	A, B, C, D, E
43	6.61	225[M + H]^+^, 247[M + Na]^+^, 207[M-H_2_O + H]^+^, 189[M + H-2H_2_O]^+^, 165[M + H-H_2_O-C_3_H_6_]^+^	223[M-H]^-^	270	Senkyunolide H	A, B, C, D, E
44	10.82	191[M + H]^+^, 173, 149, 135	189[M-H]^-^, 207[M-H + H_2_O]^-^	295, 325	*E*-ligustilide	A, B, C, D, E
45	13.74	191[M + H]^+^, 173, 149, 135	-	295, 325	*Z*-ligustilide	A, B, C, D, E
46	9.44	381[M + H]^+^, 426[M + 2Na]^+^, 191[C_12_H_15_O_2_]^+^	-	280	*Z*-ligustilide dimmer E-232	A, B, C, D, E
47	13.62	381[M + H]^+^, 426[M + 2Na]^+^, 191[C_12_H_15_O_2_]^+^	-	296	*Z,Z*^′^-3,3^′^,8,8^′^-Diligustilide	A, B, C, D
48	10.33	203[M + H]^+^, 225[M + Na]^+^	-	-	3-butylidene-7-hydroxyphthalide	A, B, C, D, E
49	8.04	189[M + H]^+^, 171[M-H_2_O + H]^+^, 153[M-2H_2_O + H]^+^, 117[M + 3H-H_2_O-CO-C_2_H_4_]^+^	187[M-H]^-^	260, 310	*E*-Butylideniphthalide	A, B, C, D, E
50	9.31	189[M + H]^+^, 161[M-CO + H]^+^, 133[M + H-CO-C_2_H_4_]^+^	187[M-H]^-^, 205[M-H + H_2_O]^-^	260, 310	*Z*-Butylidenephthalide	A, B, C, D, E
51	12.90	381[M + H]^+^, 426[M + H + HCOO]^+^, 236, 191	379[M-H]^-^, 424[M-H + HCOO]^-^, 397[M-H + H_2_O]^-^	282	Angelicide	A
52	13.65	381[M + H]^+^, 403[M + Na]^+^, 426[M + 2Na]^+^, 191[C_12_H_15_O_2_]^+^	379[M-H]^-^, 411	284	Riligustilide	A
53	14.70	381[M + H]^+^, 403[M + Na]^+^, 191[C_12_H_15_O_2_]^+^	-	280	Tokinolide B	A
54	12.60	381[M + H]^+^, 399[M + H_2_O + H]^+^, 191[C_12_H_15_O_2_]^+^	379[M-H]^-^, 397[M-H + H_2_O]^-^	230, 276	Levistolide A	A, B, C, D, E
55	3.01	613[M + H]^+^, 635[M + Na]^+^, 451, 433, 163, 144	611[M-H]^-^, 543, 353, 191	227	Hydroxysafflor yellow A	B
56	4.01	625[M + H]^+^, 593, 481, 433, 319, 301, 197	623[M-H]^-^, 611, 525, 479, 395	232	Isorhamnetin-3-*O*-nehesperridin	B
57	2.56	803[M + H]^+^, 789, 627, 325	801[M-H]^-^, 787, 593, 515, 236	230, 269, 328	6-hydroxy-keampferol −3, 6-*O*-7-*O*-glucuronide	B
58	3.85	627[M + H]^+^, 481, 319, 197	625[M-H]^-^, 525, 479, 449	232	6- hydroxy-keampferol −3, 6 – *O* – glucoside	B
59	5.13	595[M + H]^+^, 617[M + Na]^+^, 449, 287	593[M-H]^-^, 515, 449, 341, 193	241,265,330	Safflor yellow A	B
60	7.29	303[M + H]^+^	301[M-H]^-^	275	Queretin	D
61	4.81	625[M + H]^+^, 317, 479	623[M-H]^-^, 525, 315	253, 351	Isohammetin-3-*O*-neohesperidoside	D
62	5.25	625[M + H]^+^, 370, 356, 325, 317	623[M-H]^-^, 515, 315, 309, 279, 181	244, 332	Isohammetin-3-*O*-rutinoside	D
63	4.40	771[M + H]^+^, 793[M + Na]^+^, 625[M + H-rha]^+^, 479[M + H-2rha]^+^, 317[M + H-3rha]^+^	769[M-H]^-^, 525, 449, 327	253, 353	Typhaneoside	D
64	12.10	287[M + H]^+^	285[M-H]^-^	279	Keampferol	D
65	5.64	317[M + H]^+^	315[M-H]^-^	230, 273	Isohamnetin	D
66	4.90	463[M + H]^+^, 352, 322	461[M-H]^-^, 285, 193	235, 323	Scutellarin	E
67	6.33	447[M + H]^+^, 336, 352, 271	445[M-H]^-^, 891, 269	216, 277, 316	Baicalin	E
68	7.48	461[M + H]^+^, 285	459[M-H]^-^, 919, 283	220, 273	Wogonoside	E
69	8.83	271[M + H]^+^	269[M-H]^-^	275, 322	Baicalein	E
70	10.46	285[M + H]^+^	283[M-H]^-^, 268[M-H-CH_3_]^-^	274	Wogonin	E
71	7.19	461[M + H]^+^, 285	459[M-H]^-^, 283, 175	271, 310	Oroxylin-A-glucuroside	E
72	10.66	315, 373, 283	313[M-H]^-^, 375, 285	271	6-dimethoxy-wogonin	E
73	0.64	136[M + H]^+^	134[M-H]^-^		Tetramethylpyrazine	A, B, C, D, E
74	5.36	356[M + H]^+^, 338[M + H-H_2_O]^+^, 312[M + H-CO_2_]^+^	354[M-H]^-^	239, 297	Tetrahydropalmatin	C, D, E
75	4.56	342[M + H]^+^, 311, 193, 179	-	237, 278	Tetrahydrocolumbamine	C, D
76	5.71	354[M + H]^+^, 376[M + Na]^+^, 340, 320	352[M-H]^-^	238, 285	Protopine	C, D
77	5.26	370[M + H]^+^, 356[M + H-CH_2_]^+^, 327[M + H-CH_3_-CO]^+^, 326[M + H-CO_2_]^+^	368[M-H]^-^	245, 330	Allocryptopine	C, D, E
78	5.40	324[M + H]^+^, 338, 356	322[M-H]^-^	-	Tetrahydrocoptisine	C, D, E
79	6.29	356[M + H]^+^, 207	354[M-H]^-^	-	Glaucine	C, D, E
80	6.22	336[M + H]^+^, 207	354[M-H]^-^	277	Berberine	C, D, E
81	6.48	366[M + H]^+^, 308	-	262, 332	Dehydrocorydaline	C, D, E
82	5.78	368[M + H]^+^, 352[M + H-H_2_O]^+^	366[M-H]^-^	239, 277, 331	Corydaline	C, D, E
83	6.52	339[M + H]^+^, 357[M + H + H_2_O]^+^, 320[M-H_2_O]	337[M-H]^-^	237, 285	Jatrorrhizine	C, E
84	1.44	127[M + H]^+^, 149[M + Na]^+^	125[M-H]^-^, 169[M-H + HCOO]^-^	215, 272	5-hydroxymethyl-2-furfural (5-HMF)	A

### Identification of phenolic acids

In this study, 10 phenolic acids were identified from SWDCF samples according to the t*R*, UV_λmax_, and MS fragment characteristics compared with reference compounds and the literature [16,17]. They are gallic acid (**1**), chlorogenic acid (**2**), caffeic acid (**3**), vanillic acid (**4**), ferulic acid (**5**), isoferulic acid (**6**), protocatechuic acid (**7**), coumaric acid (**8**), p-hydroxy benzoic acid (**9**), and benzoic acid (**10**), respectively. The MS characteristics (Table 2) were *m/z* 169 [M - H]^-^, *m/z* 353 [M - H]^-^, *m/z* 179 [M - H]^-^, *m/z* 167 [M - H]^-^, *m/z* 193 [M - H]^-^, *m/z* 193 [M - H]^-^, *m/z* 153 [M - H]^-^, *m/z* 163 [M-H]^-^, *m/z* 137 [M - H]^-^, and *m/z* 121 [M - H]^-^, respectively.

### Identification of monoterpene, iridoid and phenylpropanoid glycosides

In the positive and negative MS experiments, there were **16** monoterpenes compounds with a pinane skeleton were analyzed and identified (Table 2). These constituents come from herbs of *Paeoniae Radix Alba* (in SWD, THSWD, XFSWD, and QLSWD) and *Paeoniae Radix Rubra* (in SFZYD). According to the MS/MS analysis of authentic compounds, the major fragmentation mechanisms of monoterpene glycosides were concluded. In the positive MS experiments, all monoterpene glycosides were ionized as sodiated molecules. The diagnostic ions of this type of compounds were the loss of glucosyl group, aglycone group, benzoyl group, which lead to the occurrence of ions at *m/z* 185, 219 or 121. Based on fragmentation patterns, the compounds **14**, **16**, and **18** were identified albiflorin, paeoniflorin, and isopaeoniflorin with MS characteristics of *m/z* 481 [M + H]^+^, *m/z* 503 [M + Na]^+^, and *m/z* 481 [M + H]^+^ by comparing to reference compounds. And compound **18** had a typical ion of *m/z* 525 [M + HCOO]^-^ at ES^-^ mode.

Structures of compounds **17**, **19**, **22**, **24** and **25** were deduced from their characteristic UV and MS spectra and fragmentation patterns. According to the literature [18-20], compounds **17**, **19** and **22** were tentatively identified as oxypaeoniflorin, galloylpaeoniflorin and mudanpioside I, respectively. The fragmentation patterns of compounds **24** and **25** were similar with those of monoterpene glycosides. In the ESI-MS/MS experiment, the diagnostic ions of *m/z* 585 [M + H]+, *m/z* 463 [M + H - benzoyl]+, *m/z* 179 [M + H – benzoyl - glu]+, *m/z* 151 [M + H - benzoyl – glu -CO] + at, and *m/z* 629 [M + HCOO]- at ES- mode. According to the literature [17], the compound 24 (tR = 7.90 min), 25 (tR = 8.09 min) were tentatively identified as benzoylalbiflorin, and isobenzoylpaeoniflorin, respectively.

Compounds **13**, **15**, **23**, and **28** were identified as sulfonates of monoterpene glycosides. Compound **13** was C_23_H_28_O_13_S and had ions of *m/z* 495 [M-CH_2_OH-H_2_O]^-^, 461 [M-SO_2_-H_2_O-H]^-^, 341 [M-SO_2_-C_7_H_5_O- 2OH]^-^ and 243 [M-glu- benzoyl-H_2_O-CH_3_]^-^. Compound **13** was plausibly identified as paeoniflorin sulfonate [21]. With the similar MS characteristics, compounds **15, 23, 28** were tentatively identified as galloylpaeoniflorin sulfonate, benzoypaeoniflorin sulfonate, isomaltopaeoniflorin sulfonate [18-20].

Compounds **11**, **12**, **20, 27** and **30** all exhibited glucosyl group. According to the MS fragments and UV_λmax_ documented in the literature [22], compounds **11**, **12, 20, 27** and **30** were tentatively identified as 1-O-*β*-D- glucopyranosyl –paeonisuffrone, 1^′^ -O -galloylsucrose, pentagalloylglucose, isomaltopaeoniflorin or 6^′^-O-*β*-D- glucopyranosylalbiflorin, and 6-O-*β*-D-glucopyranosyl lactinolide, respectively.

Three phenylpropanoid glycosides were detected and tentatively assigned as acteoside/isoacteoside / forsythoside A (**21**), cistanoside A or jionoside A1/A2 (**26**), decaffeoyl-verbascoside (**29**) according to the literatures [21,23-25]. The MS spectrums were listed in Table 2.

The *Rehmanniae Radix* is rich sources of iridoid glycosides [21,23-25]. At the positive and negative MS experiments, **9** iridoid compounds were analyzed and identified in SWDCF expcept SFZYD (Table 2). By comparing the t*R*, UV_λmax_ and MS characteristics with reference compounds and literature [23,26], they were identified as rehmannioside D (**31**), melittoside (**32**), echinacoside (**33**), rehmaionoside A/B (**34**), jionoside B1/B2 (**35**), martynoside isomer (**36**), martynoside (**37**), rehmapicroside (**38**), and rehmapicrogenin (**39**), respectively. And amygdalin (**40**) was detected and identified in THSWD.

### Identification of lactones

*Angelicae sinensis Radix* and *Chuanxiong Rhizoma* are rich sources of lactones or phthalide compounds [16,26-28]. In the positive ion mode, **14** lactones were analyzed and identified by comparing with reference compounds and literature data [27,29]. Compounds **41, 42**, and **43** were tentatively identified as senkyunolide J (**41**), senkyunolide I (**42**), and senkyunolide H (**43**), respectively.

Compounds **44**, **45**, **49** and **50** were isomers of ligustilide and butylideniphthalide. The *Z*-Ligustilide and *Z*-Butylidenephthalide were adopted as references, the compounds **44** and **45** were isomers and identified as *E*-ligustilide and *Z*- ligustilide with MS ion *m/z* 191 [M + H]^+^; the compounds **49** and **50** were isomers and identified as *E*-Butylideniphthalide and *Z*-Butylidenephthalide with MS ion of *m/z* 189[M + H]^+^, respectively [22].

Six dimmer compounds were identified from SWDCF possessing the same MS fragments ion *m/z* 381 [M + H]^+^. According to the literature data [21,28,30], these compounds were tentatively identified as *Z*-ligustilide dimmer E-232 (**46**), *Z*, *Z*^′^-3,3^′^,8,8^′^-Diligustilide (**47**), angelicide (**51**), riligustilide (**52**), tokinolide B (**53**), and levistolide A (**54**), respectively.

### Identification of flavonoids

Among 18 flavonoids identified from SWDCF, there were eight flavonoid aglycones including quercetin (**60**), isorhamnetin (**65**), keampferol (**64**), scutellarin (**66**), baicalein (**69**), wogonin (**70**), 6-dimethoxy-wogonin (**72**), and oroxylinA (**71**). The flavonoid glycosides were identified by comparing the t*R*, UV_λmax_, and the MS fragments characteristics to the standard substances.

Compounds **61**, **62**, and **63** contained fragment ion *m/z* 317 (Table 2). The MS/MS of *m/z* showed fragments including *m/z* 287, 273, 153, and 123. These data were consistent with those in the literatures [31,32]. The aglucone was identified as isohamnetin. The three flavonoid glycosides were identified as isohamnetin-3-O-neohesperidoside (**61**), isohamnetin-3-O-rutinoside (**62**), and typhaneoside (**63**), respectively.

Hydroxysafflor yellow A (**55**) and safflor yellow A (**59**) were detected in THSWD from *Carthami Flos*. Compound **59** possessed MS ions characteristics of *m/z* 595[M + H]^+^ and *m/z* 617[M + Na]^+^ at ES^+^ mode and *m/z* 593[M-H]^-^ at ES^-^ mode. Compound 55 had diagnostic ions of *m/z* 613[M + H]^+^, and *m/z* 635[M + Na]^+^ at ES^+^ mode and *m/z* 611[M-H]^-^ at ES^-^ mode. Baicalin (**67**) and wogonoside (**68**) were identified from QLSWD [33,34].

### Identification of alkaloids

The alkaloids compounds derived mainly from *Chuanxiong Rhizoma*, *Corydalis Rhizoma*, and *Coptidis Rhizoma*. At the positive ion mode, **11** alkaloids constituents were analyzed and identified from SWDCF by comparing with reference compounds and literature data [35]. Compound **74** with MS characteristics of *m/z* 356 [M + H]^+^, 338 [M + H-H_2_O]^+^, and 312 [M + H-CO_2_]^+^ was identified as tetrahydropalmatine compared with the reference standard. Compounds **73, 76, 78, 80, 81, 82,** and **83** were detected and identified from XFSWD, SFZYD, and QLSWD as tetramethylpyrazine (**73**), protopine (**76**), tetrahydrocoptisine (**78**), berberine (**80**), dehydrocorydaline (**81**), corydaline (**82**), and jatrorrhizine (**83**) by comparing with the reference standards, respectively. The MS characteristics were *m/z* 136 [M + H]^+^, *m/z* 354 [M + H]^+^, *m/z* 324 [M + H]^+^, *m/z* 336 [M + H]^+^, *m/z* 366 [M + H]^+^, *m/z* 368 [M + H]^+^, and *m/z* 339 [M + H]^+^, respectively. According to the literature [35], compounds **75**, **77**, and **79** were tentatively identified as tetrahydrocolumbamine, allocryptopine, and glaucine, respectively. The MS characteristics ions were *m/z* 342 [M + H]^+^, *m/z* 370 [M + H]^+^, and *m/z* 356 [M + H]^+^, respectively. In addition, 5-hydroxymethyl-2-furfural (5-HMF) (**84**) was identified from SWD only.

UPLC-Q-TOF-MS method was employed to identify the constituents from SWDCF. In ESI-TOF-MS experiment, accurate molecular mass of the components can be obtained. As ESI was a soft ionization technique, the interface produces little fragmentation of analytes and generally forms protonated molecular ions [M + H]^+^ for positive ionization mode or [M-H]^-^ for negative ionization mode. Comparing the mass spectra of the compounds with the standards and those in the literature, the common and different components were unequivocally identified from every formula of SWDCF. These data would be provided the bioactive components for activities of different formulae.

### The markers obtained by MarkerLynx™

The principal components analysis (PCA) was done by the Waters MarkerLynx™ software. In this study, SWD, THSWD, XFSWD, SFZYD and QLSWD were injected six times each in two ESI modes. Unsupervised PCA was performed to globally evaluate the chemical consistency among these five SWDCF decoctions. The data from both positive and negative ion modes were displayed as scores plots (Additional file 1: Figures S2-1 and 2-2). The scores plots demonstrated a clear classification trend among SWDCF samples, with all the observations falling within the Hotelling T2 (0.95) ellipse, which confirmed the fact that chemical difference exists among SWDCF. The results showed that the SFZYD and QLSWD were significantly different to SWD, THSWD, and XFSWD, while SWD, THSWD, and XFSWD were close to each other. These data indicated that the chemical composition and quantity of components changed after combining SWD with different herbs.

**Figure 2:**
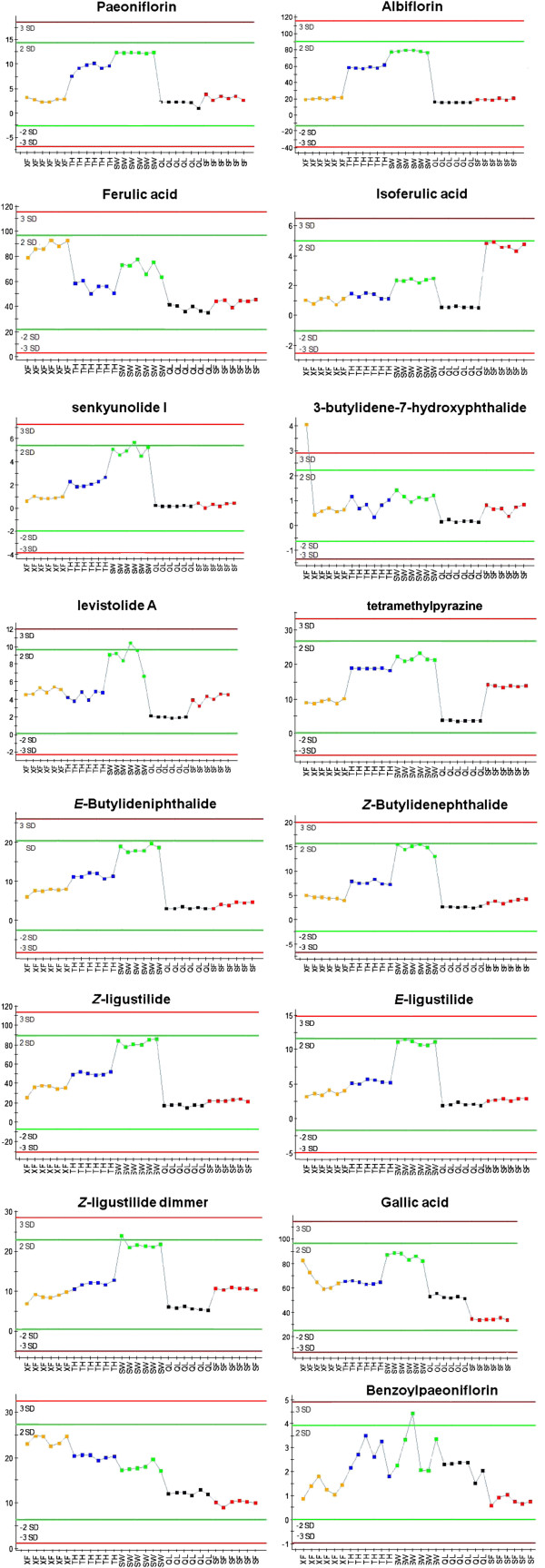
**The intensity trend plots of sixteen representative changed common components in SWDCF samples.** X axis represent for SWDCF samples (XF: XFSWD; TH: THSWD; SW: SWD; QL: QLSWD; SF: SFZYD). Y axis represent for relative quantity of target compounds.

Chemical markers were analyzed to find out the changed common components contributing most to the SWDCF. Figures 2 showed the representative mass spectra of sixteen common and the greatest change compounds. According to the intensity trends of fragment ions from five decoctions samples, the intensity of the common chemical markers (paeoniflorin **16**, albiflorin **14**, ferulic acid **5**, isoferulic acid **6**, senkyunolide I **42**, 3-butylidene-7-hydroxyphthalide **48**, levistolide A **54**, tetramethylpyrazine **73**, *E*-Butylideniphthalide **49**, *Z*-Butylidenephthalide **50**, *Z*-ligustilide **45**, *E*-ligustilide **44**, *Z*-ligustilide dimmer **46**, gallic acid **1**, chlorogenic acid **2**, benzoylpaeoniflorin **24**) were different in every formula of SWDCF.

The peak areas of components **16** (t*R* 5.80 min, *m/z* 481.1750), **14** (t*R* 3.90 min, *m/z* 481.1710), **42** (t*R* 6.26 min, *m/z* 225.1144), **54** (t*R* 12.60 min, *m/z* 399.2193), **73** (t*R* 0.64 min, *m/z* 136.0622), **49** (t*R* 8.08 min, *m/z* 189.0919), **50** (t*R* 9.27 min, *m/z* 189.0919), **46** (t*R* 13.80 min, *m/z* 191.1049), **45** (t*R* 10.82 min, *m/z* 191.1079), **44** (t*R* 9.44 min, *m/z* 191.1073) were higher in SWD than other formulae. XFSWD possessed greater peak area of compound 2 and 5, while SFZYD possessed high content of compound 6.

### Optimization of the HPLC conditions

A small amount of acid was added into the mobile phase which could inhibit the ionization of these components to improve the peak shape and restrain the peak tailing due to the existence of acidic ingredients in SWDCF samples. 0%, 0.1% and 0.2% aqueous formic acid and acetic acid solutions were compared. The results showed that 7 compounds could be baseline separated when 0.1% aqueous formic acid solution was selected.

**Figure 3:**
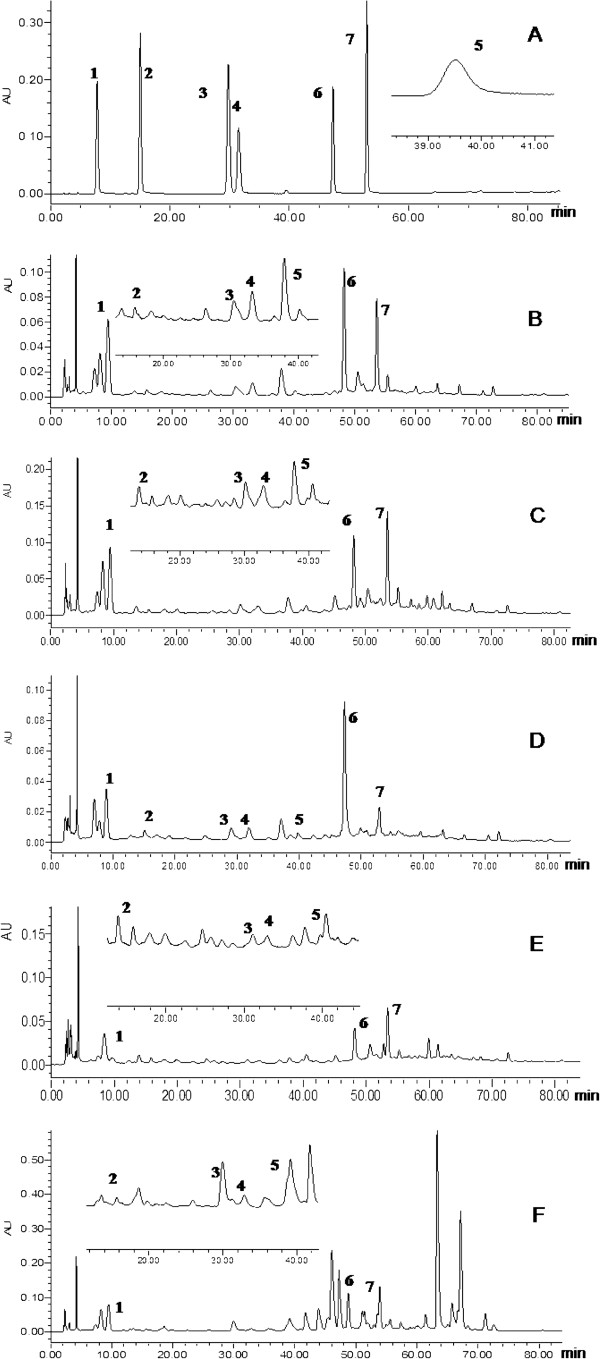
**Typical HPLC-DAD chromatograms of mixed standards and samples. A**: mixed standards; **B**: SWD; **C**: THSWD; **D**: XFSWD; **E**: SFZYD; **F**: QLSWD (**1.** gallic acid, **2.** protocatechuic acid,**3.** vanillic acid, **4.** caffeic acid, **5.** paeoniflorin, **6.** ferulic acid, **7.** senkyunolide I).

DAD detection was set at the wavelength range of 190–400 nm. For the satisfactory sensitivity, resolution and lower noise, four wavelengths at 230 nm, 260 nm, 277 nm, and 320 nm were selected for determining the different compounds in SWDCF. According to absorption curve of the tested analytes, the paeoniflorin had optimal sensitivity for detection at 230 nm, 320 nm for ferulic acid, 260 nm for gallic acid, vanillic acid, and caffeic acid, and 277 nm for senkyunolide I. Thus, a switching UV wavelength method was established by a variable-wavelength spectrophotometric detector. Under the optimized HPLC-UV conditions, the investigated analytes were well separated and detected in 85 min (Figure 3).

Prior to sample analysis the optimal process of extraction had to be investigated. According to the applied form of decoction, the SWDCF were extracted by refluxing with water for twice. Ethanol (95%) was added to the filtrates until the concentration of ethanol was adjusted to 50% and seven compounds were almost completely extracted.

### Validation of the quantitative analysis

The HPLC–DAD method for quantitation analysis was validated to determine the linearity, LOD, and LOQ. Good linear correlation and high sensitivity at these chromatographic conditions were confirmed by the correlation coefficients r^2^ > 0.9990 and *P* = 3.7501 × 10^-9^, 1.3503 × 10^-7^, 3.7553 × 10^-8^, 0, 3.7513 × 10^-7^, 3.7553 × 10^-8^, 3.7501 × 10^-9^ for gallic acid, protocatechuic acid, vanillic acid, caffeic acid, paeoniflorin, ferulic acid, senkyunolide I, respectively, within the test ranges, and the overall LODs and LOQs were in the range of 5.0–24.8 and 10.0–59.6 μg/mL, respectively (Table 3).

**Table 3:** Calibration curves, LOD and LOQ data of investigated compounds by HPLC-DAD

**Analytes**	**Calibration curves **^**a**^	***r***^***2***^	**Linear range (μg/mL)**	**LOD (μg/mL)**	**LOQ (μg/mL)**
Gallic acid	Y = 2.4 × 10^6^X-8022.9	0.9999	148.0-1480.0	7.5	14.1
Protocatechuic acid	Y = 8.0 × 10^6^X-36350	0.9994	38.4-384.0	8.6	19.2
Vanillic acid	Y = 2.6 × 10^6^X-18490	0.9997	32.0-320.0	5.0	10.0
Caffeic acid	Y = 3.5 × 10^6^X-25506	1.0000	35.6-356.0	6.5	13.6
Paeoniflorin	Y = 1.1 × 10^6^X + 160000	0.9990	476.0-4760.0	24.8	59.6
Ferulic acid	Y = 1.1 × 10^7^X-1.2 × 10^5^	0.9997	136.0-1360.0	6.2	12.4
Senkyunolide I	Y = 6.5 × 10^6^X-9533.5	0.9999	46.0-460.0	6.3	12.6

^a^ y is the logarithmic value of peak area and x is the logarithmic value of the reference compound’s concentration (μg/ml).

As shown in Table 4, the intra- and inter-day precisions, repeatability and stability of the seven analytes were less than 3%. The overall recoveries lay between 92.20% and 104.60% with RSD less than 3.54% for seven components in all samples. These results indicated that the HPLC fingerprint chromatograms had a good repeatability, precision, accuracy, and recovery (Tables 4 and 5) and the developed HPLC-DAD method was a reliable and useful method for assessment of SWDCF.

**Table 4:** Precision, repeatability of seven analytes

**Analytes**	**Precision (RSD,%)**		**Repeatability (RSD,%; n = 6)**				
	**Intraday (n = 6)**	**Interday (n = 6)**	**SWD**	**SFZYD**	**XFSWD**	**THSWD**	**QLSWD**
Gallic acid	1.75	1.78	1.81	1.26	2.84	3.32	1.31
Protocatechuic acid	0.76	1.21	3.16	1.90	2.72	4.18	3.19
Vanillic acid	1.31	1.55	1.79	1.05	2.26	3.97	2.80
Caffeic acid	0.98	0.97	2.27	2.47	1.60	4.52	3.04
Paeoniflorin	1.06	2.01	2.72	3.58	2.38	3.96	2.19
Ferulic acid	0.88	1.05	2.19	3.01	1.26	2.95	2.22
Senkyunolide I	1.16	1.12	2.22	3.97	1.43	1.82	3.75

**Table 5:** Stability and recovery of seven analytes

**Analytes**	**Stability (RSD,%; n = 6)**					**Recovery (%; n = 6)**									
	**A**	**B**	**C**	**D**	**E**	**SWD**		**THSWD**		**XFSWD**		**SFZYD**		**QLSWD**	
						**Mean**	**RSD (%)**	**Mean**	**RSD (%)**	**Mean**	**RSD (%)**	**Mean**	**RSD (%)**	**Mean**	**RSD (%)**
Gallic acid	6.36	3.34	7.13	5.16	1.03	98.1	2.71	98.9	1.32	97.8	2.32	101.2	3.12	99.7	1.44
Protocatechuic acid	5.79	5.46	6.18	4.0	4.62	101.2	3.32	99.8	1.43	97.6	1.18	103.7	3.54	102.7	1.98
Vanillic acid	3.14	3.68	6.25	8.80	2.19	96.2	1.39	97.3	1.79	102.3	1.25	92.2	2.81	103.1	2.32
Caffeic acid	6.61	5.10	8.50	2.47	7.87	103.8	1.48	95.8	3.24	98.3	2.50	98.6	2.44	98.2	3.16
Paeoniflorin	6.13	3.79	7.37	8.36	4.07	97.1	3.25	98.2	2.77	95.4	1.15	94.0	1.36	95.8	2.17
Ferulic acid	4.54	3.74	1.31	7.65	2.10	99.7	1.22	103.2	1.94	99.3	1.43	97.5	2.65	104.6	1.89
Senkyunolide I	6.87	2.82	2.47	8.91	4.07	96.9	2.31	98.1	2.13	98.1	2.86	94.0	1.91	98.1	2.34

### Sample analysis

The HPLC-DAD method was then subsequently applied to simultaneously determine the chemical markers including gallic acid, protocatechuic acid, vanillic acid, caffeic acid, paeoniflorin, ferulic acid, senkyunolide I in SWDCF samples. The results (Table 6) showed there were remarkable differences among the contents of the chemical markers analyzed in different samples. Paeoniflorin (**5**) was found to be a predominant constituent in both of QLSWD and SFZYD, while the lowest contents in SWD except XFSWD, suggesting that the active compounds of paeoniflorin was dissolved increasedly after the SWD combined with other herbs. The contents of senkyunolide I in SWDCF were decreased except SFZYD varing from 31.30 to 84.70 mg/g. The phenolic acids including gallic acid, caffeic acid, ferulic acid, vanillic acid, and protocatechuic acid were increased significantly except in XFSWD.

**Table 6:** Contents of seven investigated compounds in SWDCF

**Analytes**	**Contents of analyst (mean ± SD; n = 3; μg/g)**				
	**SWD**	**THSWD**	**XFSWD**	**SFZYD**	**QLSWD**
Gallic acid	316.00	421.20	143.50	436.00	729.80
Protocatechuic acid	6.50	11.60	5.40	2.46	4.05
Vanillic acid	18.40	33.90	13.80	90.70	44.00
Caffeic acid	58.20	69.70	31.30	67.30	84.70
Paeoniflorin	2050.00	3430.00	790.00	3840.00	5140.00
Ferulic acid	120.00	166.70	81.30	225.70	310.70
Senkyunolide I	180.00	30.00	70.00	210.00	80.00

## Conclusion

The chemical profiles with 84 components in SWDCF, including monoterpene glycosides, acetophenones, galloyl glucoses, even some isomers in the complex system were characterized by UPLC–QTOF–MS/MS.

## Abbreviations

SWDCF: Siwu decoction categorized formulae; UPLC - QTOF - MS /MS: Ultra-high performance liquid chromatography coupled with time-of-flight mass spectrometry; CM: Chinese medicine; CF: Categorized formulae; SWD: Siwu decoction; THSWD: Taohong Siwu decoction; PD: Primary dysmenorrheal; XFSWD: Xiangfu Siwu decoction; SFZYD: Shaofu Zhuyu decoction; QLSWD: Qinlian Siwu decoction; ESI: Electrospray ionization

## Competing interest

The authors declare that they have no competing interest.

## Authors’ contribution

JD designed the experiment. SS performed the experiments, analyzed the data and wrote the manuscript. WC, and ES performed the experiments. WZ analyzed the data. YT revised the manuscript. All authors read and approved the final version of the manuscript.

## Supplementary Material

Additional file 1: Figure S1 Typical BPI chromatogram of SWDCF (A) SWD, (B) THSWD, (C) XFSWD, (D) SFZYD, and (E) QLSWD. Figure S1-1 ESI^+^; Figure S1-2 ESI. PCA model results between SWDCF samples. (A, ESI^+^; B, ESI^-^). Click here for file
