# Supplementary material for: Chemical fingerprinting and quantitative constituent analysis of Siwu decoction categorized formulae by UPLC-QTOF/MS/MS and HPLC-DAD
Source: Chin Med. 2013 Mar 1;8:5. doi: 10.1186/1749-8546-8-5 (PMC3602048; doi:10.1186/1749-8546-8-5)
Supplement: Additional file 1: Figure S1 — Typical BPI chromatogram of SWDCF (A) SWD, (B) THSWD, (C) XFSWD, (D) SFZYD, and (E) QLSWD. Figure S1-1 ESI+; Figure S1-2 ESI. PCA model results between SWDCF samples. (A, ESI+; B, ESI-). [file 1749-8546-8-5-S1.pdf]

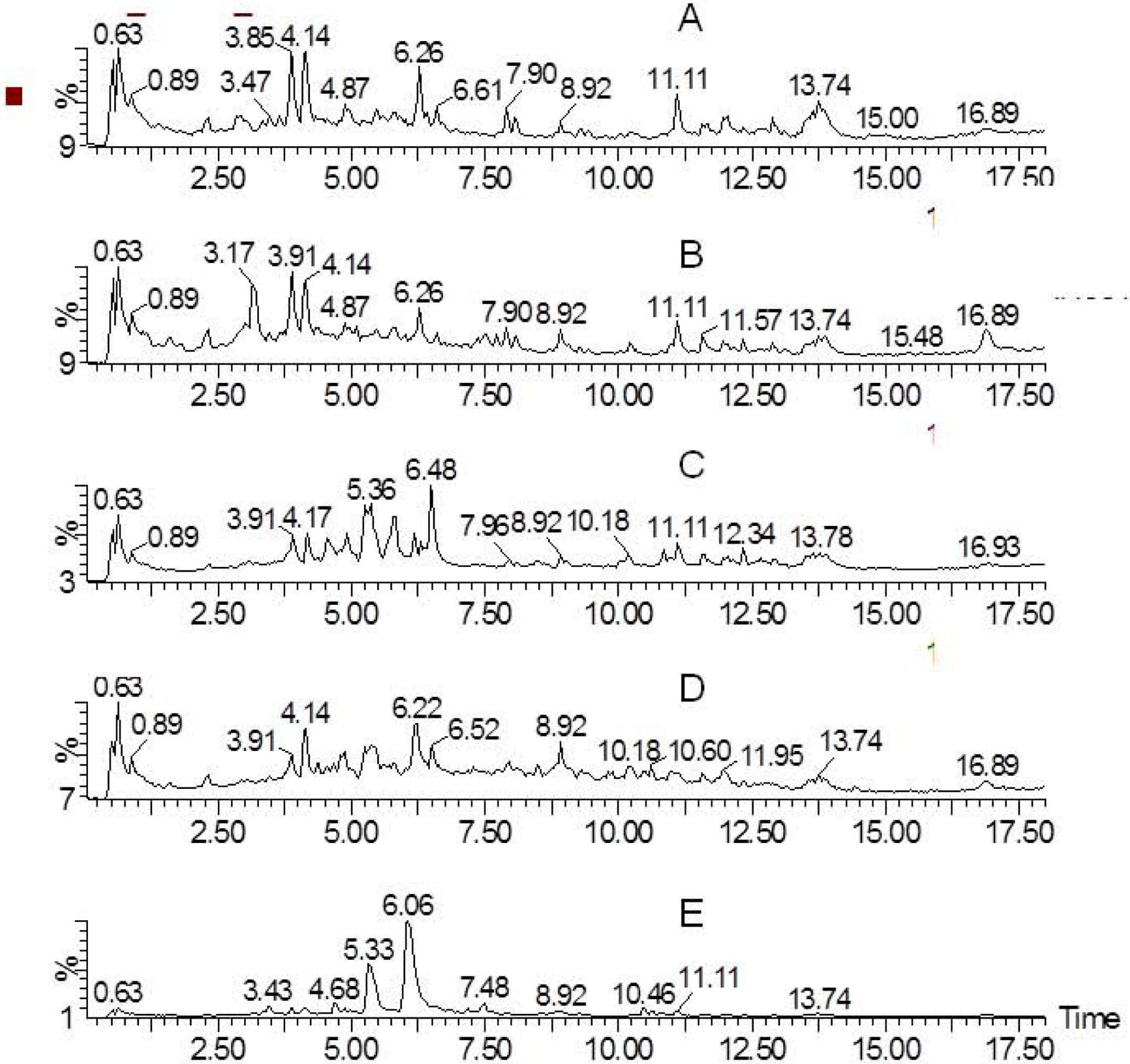

A: SWD, B: THSWD, C: XFSWD, D: SFZYD, E: QLSWD

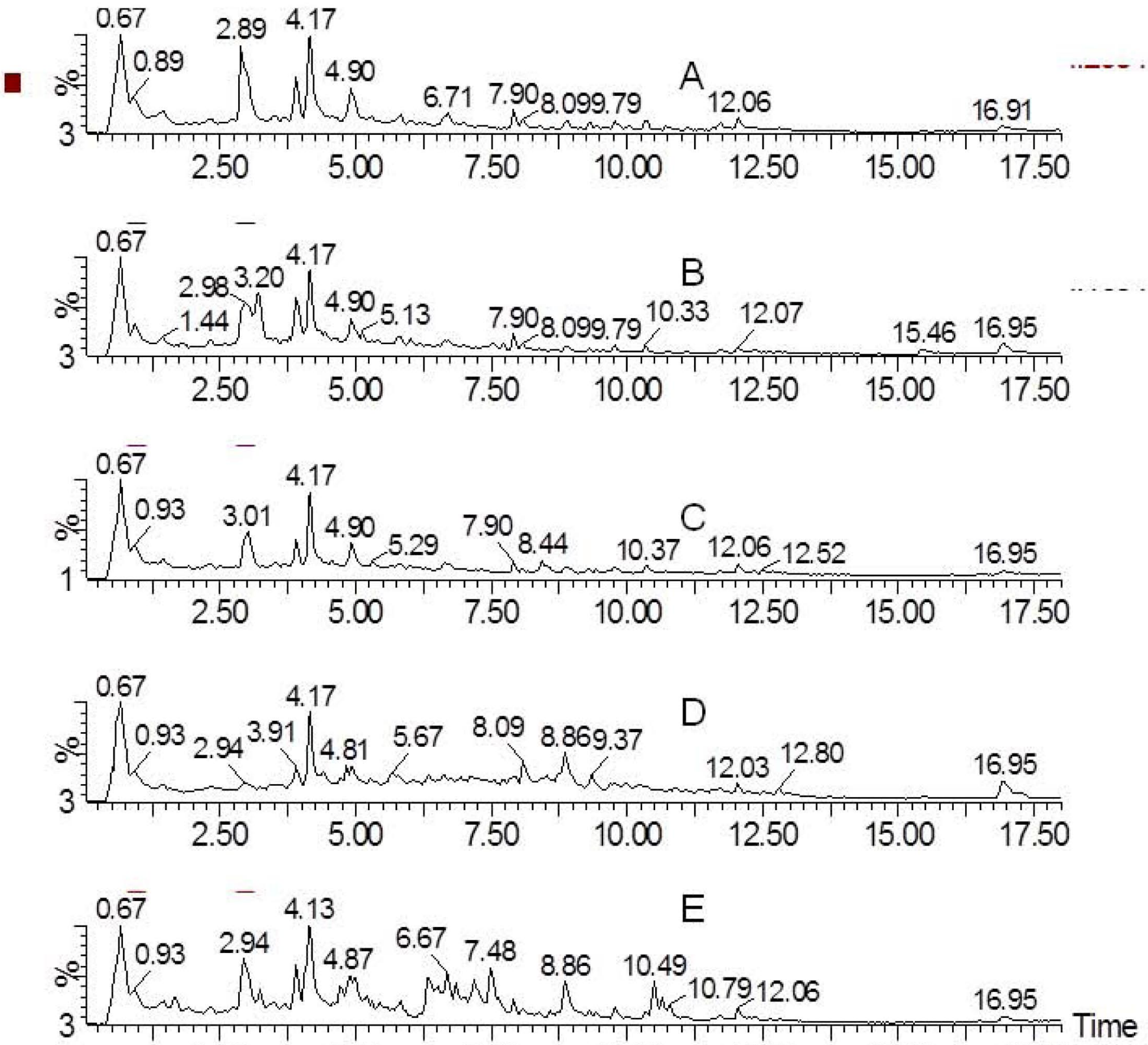

A: SWD, B: THSWD, C: XFSWD, D: SFZYD, E: QLSWD

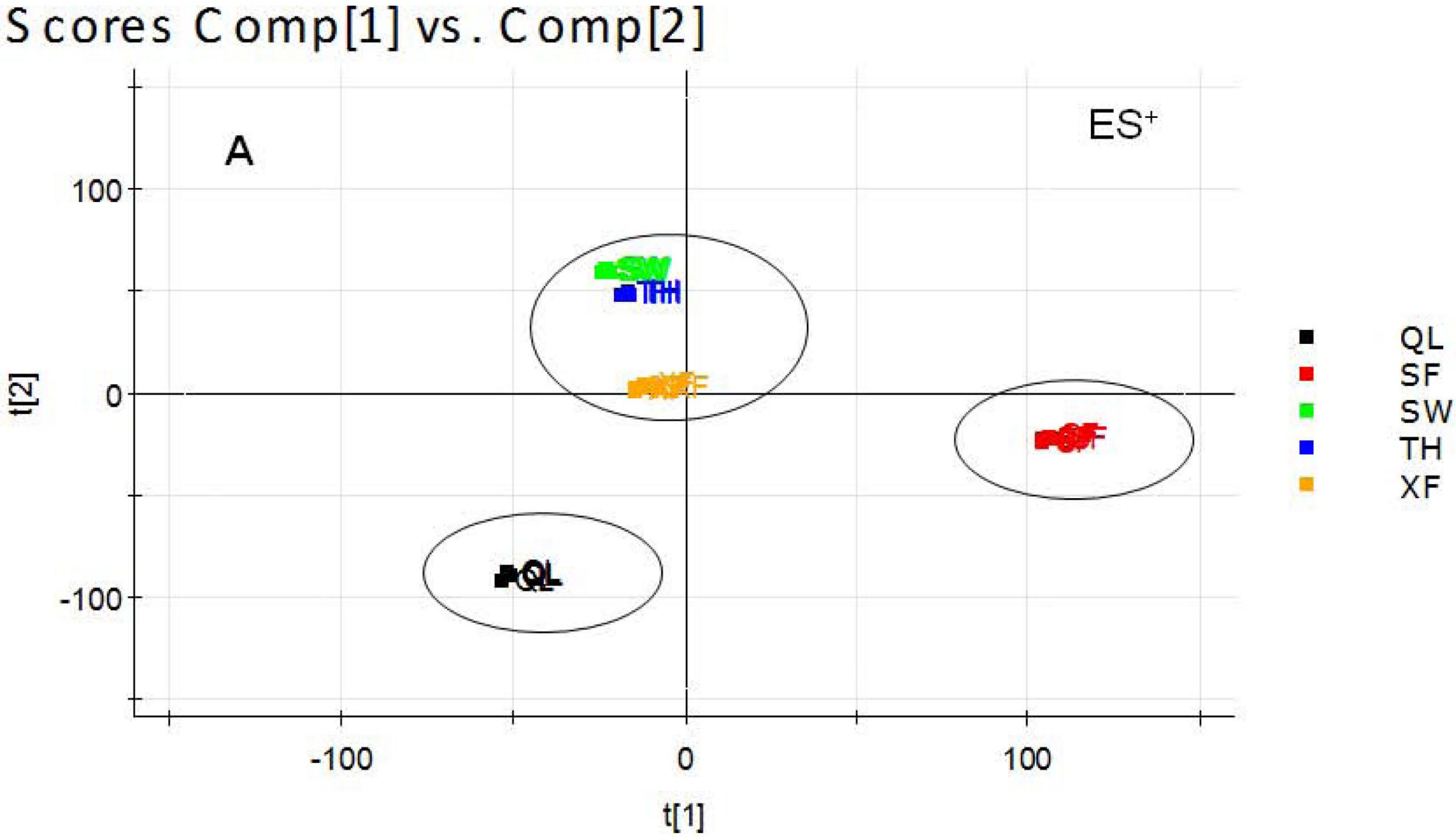

Scores Comp[1] vs. Comp[2]
A
ES+
100
0
-100
-100
0
100
t[1]
t[2]
SW
TH
XF
SF
QL
QL
SF
SW
TH
XF

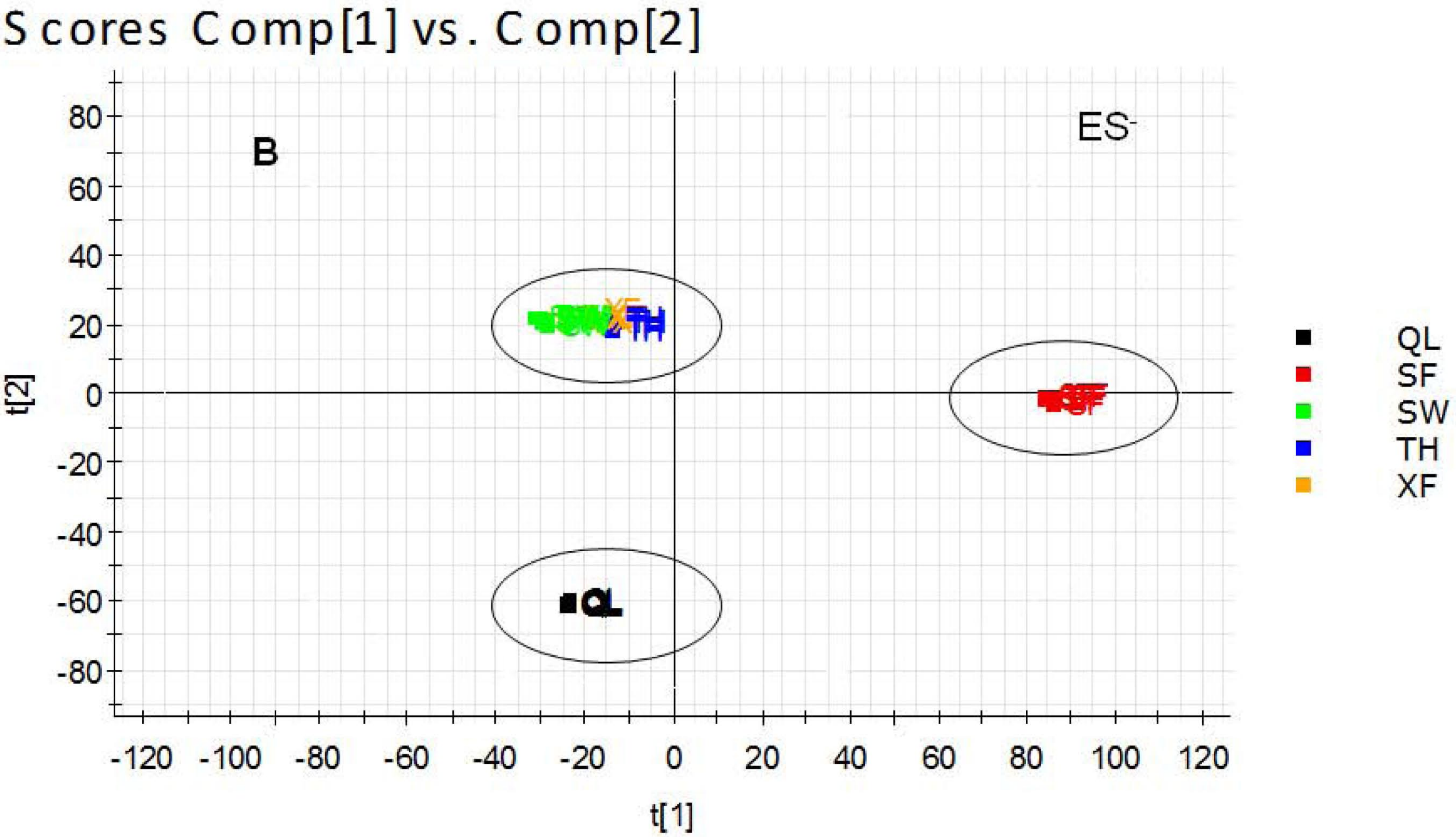
S cores C omp[1] vs. C omp[2]
B
ES-
80
60
40
20
0
-20
-40
-60
-80
t[2]
-120
-100
-80
-60
-40
-20
0
20
40
60
80
100
120
t[1]
QL
SF
SW
TH
XF
